# Predictors of quality of care in mental health supported accommodation services in England: a multiple regression modelling study

**DOI:** 10.1186/s12888-018-1912-7

**Published:** 2018-10-20

**Authors:** Christian Dalton-Locke, Rosie Attard, Helen Killaspy, Sarah White

**Affiliations:** 1 Division of Psychiatry, University College London, Maple House, 149 Tottenham Court Road, London, W1T 7NF UK; 2 Population Health Research Institute, St George’s, University of London, Cranmer Terrace, London, SW17 0RE UK

**Keywords:** Mental health, Supported accommodation, Quality assessment, Quality of care, Predictors of quality, Multiple regression

## Abstract

**Background:**

Specialist mental health supported accommodation services are a key component to a graduated level of care from hospital to independently living in the community for people with complex, longer term mental health problems. However, they come at a high cost and there has been a lack of research on the quality of these services. The QuEST (Quality and Effectiveness of Supported tenancies) study, a five-year programme of research funded by the National Institute for Health Research, aimed to address this. It included the development of the first standardised quality assessment tool for supported accommodation services, the QuIRC-SA (Quality Indicator for Rehabilitative Care – Supported Accommodation). Using data collected from the QuIRC-SA, we aimed to identify potential service characteristics that were associated with quality of care.

**Methods:**

Data collected from QuIRC-SAs with 150 individual services in England (28 residential care, 87 supported housing and 35 floating outreach) from four different sources were analysed using multiple regression modelling to investigate associations between service characteristics (local authority area index score, total beds/spaces, staffing intensity, percentage of male service users and service user ability) and areas of quality of care (Living Environment, Therapeutic Environment, Treatments and Interventions, Self-Management and Autonomy, Social Interface, Human Rights and Recovery Based Practice).

**Results:**

The local authority area in which the service is located, the service size (number of beds/places) and the usual expected length of stay were each negatively associated with up to six of the seven QuIRC-SA domains. Staffing intensity was positively associated with two domains (Therapeutic Environment and Treatments and Interventions) and negatively associated with one (Human Rights). The percentage of male service users was positively associated with one domain (Treatments and Interventions) and service user ability was not associated with any of the domains.

**Conclusions:**

This study identified service characteristics associated with quality of care in specialist mental health supported accommodation services that can be used in the design and specification of services.

## Background

Specialist mental health supported accommodation services support an estimated 60,000 people in England [1, 2] and form an essential component of the whole-system care pathway for people with complex, longer term mental health problems [3]. They provide a graduated level of support for people discharged from hospital and are usually found in countries which have gone through a process of deinstitutionalisation i.e. the closure of asylums and development of community care.

In England, these services can be classified into three main types [4]: (1) residential care homes, which are staffed 24 h per-day, provide day-to-day necessities such as meals and medication administration, and are usually not time-limited; (2) supported housing services, which provide time-limited tenancies with shared or self-contained flats with staff on-site up to 24 h per-day; and (3) floating outreach services, which provide visiting (off-site) support to service users in permanent (not time-limited) tenancies. Most service users in residential care and supported housing have a diagnosis of psychosis compared to around half of those using floating outreach (the remainder have common mental disorders such as depression or anxiety). Service users in residential care have the highest level of needs followed by supported housing and floating outreach [3].

Despite the large number of people using mental health supported accommodation services and the associated costs, these services have been under researched. The Quality and Effectiveness of Supported Tenancies for people with mental health problems project (QuEST), a five-year research programme that commenced in 2012 funded by the National Institute for Health Research (NIHR) (Application RP-PG-0610-10,097), aimed to address this evidence gap. It included: the adaptation of a standardised quality assessment tool (the Quality Indicator for Rehabilitative Care – QuIRC) for use in supported accommodation services; a national survey of supported accommodation services and their service users across England [3]; a naturalistic, prospective cohort study investigating 30-month outcomes for service users; and a feasibility study to assess whether it is possible to carry out a large scale randomised control trial comparing two supported accommodation models (supported housing and floating outreach).

The QuIRC was developed to assess the quality of care in psychiatric and social care facilities for adults with longer term mental health problems across Europe and its development has been described elsewhere [5]. In summary, its content was derived from triangulation of the results of a systematic literature review [6], international Delphi exercise [7] and review of care standards in each of ten participating European countries. Item scores are collated to assess seven domains of care: the Living (built) Environment; the Treatments and Interventions provided; the Therapeutic Environment (culture of the unit); the promotion of Self-Management and Autonomy; the promotion of Social Interface with the community and family/friends; the protection of Human Rights; the implementation of Recovery Based Practice. Examples of questions and the domains they score on are presented in Table 1. Some questions score on more than one domain, for example, question ‘Roughly what percentage of your residents/service users will be assisted to vote in the next political election?’ scores for Social Interface, Human Rights and Recovery Based Practice.

**Table 1:** QuIRC-SA domains and example questions^a^

Domain	Example question 1 [and response options/format]	Example question 2 [and response options]
Living Environment	What do you think of the general condition of the building outside? (Select one) [Very poor condition/Quite poor condition/Acceptable condition/Quite good condition/Very good condition]	What do you think of the general décor indoors? (Select one) [Very poor condition/Quite poor condition/Acceptable condition/Quite good condition/Very good condition]
Therapeutic Environment	How hopeful are you that the majority of your current residents/service users will show improvement in their general functioning over the next 2 years? [Number]	We know it is not always possible to keep staff up to date with new developments but we are interested in knowing what types of training the staff in your project/service have received. In which of the following areas have your staff received FORMAL training in the last 12 months and how many staff members received this training? [Yes / No to several areas in mental health (e.g. comunication skills, mental health awareness), and number of staff that received training]
Treatments and Interventions	How many of your residents/service users regularly take part in programmed activities in the project/service? [Number]	Do you use individual care-plans for all residents/service users? [Yes / No]
Self-Management and Autonomy	Do residents/service users who have legal capacity have full control over their finances? [Yes / No]	Is there a process for supporting service users to manage their own medication? [Yes/No]
Social Interface	How many of your residents/service users have regular contact with non-service user friends? [Number]	Roughly what percentage of your residents/service users will be assisted to vote in the next political election? [Percentage]
Human Rights	Are patient’s/residents’ records kept in a locked environment (e.g. locked staff office, locked cabinet, password-protected computer)? [Yes/No]	Do you have a formal complaints procedure? [Yes / No]
Recovery Based Practice	In general, how would you say your project/service mostly aims to assist residents/service users? (Select one) [To assist residents/service users to gain and regain skills to live more independently/To provide residents/service users with the care they need because of their disability / Both equally]	How often do you have meetings where staff and residents/service users discuss the running of the project/service? (Select one) [Never/Every 7 to 12 months/Every 4 to 6 months/Every 2 to 3 months/Every 2 to 6 weeks/Weekly or more than weekly]

^a^Please note that some questions score on to more than one domain

The QuIRC has good inter-rater reliability [5] and the domain scores derived are positively associated with service users’ experiences of care [8]. It is available as a web based application (www.quirc.eu) in the ten languages of the countries that participated in its development. Results are presented in a printable report showing the unit’s performance on each domain as a percentage on a “spider web” diagram, which also shows the average performance for similar units in the same country.

The QuIRC was adapted for supported accommodation services (QuIRC-SA) through an iterative process of consultation with relevant stakeholders in England during the QuEST Study and its psychometric properties assessed [9]. Specifically, focus groups were carried out with staff from the three main types of supported accommodation service and three expert panels were consulted (two comprised individuals with lived experience of supported accommodation services and one comprised senior professionals and policy makers with expertise in supported accommodation) to suggest appropriate amendments. The adapted tool has good psychometric properties [9]. The QuIRC-SA comprises the same seven domains as the original QuIRC but floating outreach services are not assessed on the Living Environment domain as staff visit service users in their own homes (the service does not provide the building).

The QuIRC has been used in national and international studies investigating longer term mental health services [8, 10] which have found quality of services to be positively associated with geographic location (urban/rural) and smaller, mixed sex units with an expected maximum length of stay and where there is a range of disability amongst service users.

The QuIRC-SA was used in the QuEST programme during the national survey of supported accommodation carried out in 2013–14 [3]. This involved 87 services (22 residential care, 35 supported housing, 30 floating outreach) randomly selected from 14 nationally representative areas across England. Supported housing services scored higher than residential care and floating outreach on six of the seven QuIRC-SA domains and floating outreach scored highest on the human rights domain.

In 2016, the QuIRC-SA was also completed with four supported housing service managers during the feasibility study component of the QuEST programme, and with 80 supported housing services as part of a national survey of staff morale being undertaken by the QuEST team. It was also completed with managers of 54 supported accommodation services in the London boroughs of Camden and Islington as part of a local audit in 2016 (11 residential care, 34 supported housing, nine floating outreach).

We aimed to use these four sources of QuIRC-SA data to investigate service characteristics associated with quality of care in mental health supported accommodation services in England.

## Methods

### Sample

A total of 195 QuIRC-SAs were completed for 150 specialist mental health supported accommodation services across England between October 2013 and January 2017. Where a service had completed the QuIRC-SA more than once (45 services), only the most recently completed QuIRC-SA was retained for the current analysis. The final sample comprised 28 residential care, 87 supported housing and 35 floating outreach services. Table 2 shows the data sources for this study.

**Table 2:** Data sources and number of QuIRC-SAs completed and retained in the current analysis

Data source	Residential care			Supported housing			Floating outreach			All services		
	N	Services completed > one QuIRC-SA	QuIRC-SAs retained^a^	N	Services completed > one QuIRC-SA	QuIRC-SAs retained^a^	N	Services completed > one QuIRC-SA	QuIRC-SAs retained^a^	N	Services completed > one QuIRC-SA	QuIRC-SAs retained^a^
National survey	22	5	17	6	5	1	31	5	26	59	5	44
Staff morale survey	0	0	0	79	0	79	0	0	0	79	0	79
Local audit	11	0	11	4	0	4	9	0	9	24	0	24
Feasibility trial	0	0	0	3	0	3	0	0	0	3	0	3
TOTAL	33	5	28	92	5	87	40	5	35	165	5	150

^a^Where services completed more than one QuIRC-SA, only the most recently completed QuIRC-SA was retained for the regression models

### Data analysis

The sample of 150 services provided 80% power to estimate the association of six service characteristics with each of the seven QuIRC-SA domain scores with a small to medium effect size (of 0.35) at a significance level of 0.7% [11]. This reduced significance level accounts for the multiple hypothesis testing conducted (seven regression models, one for each domain of the QuIRC-SA).

The following six service characteristics were investigated for their association with domain scores and entered as independent variables into multiple linear regression models using Stata 14: (1) local authority area rank index score for the location of the service. This is a sampling index developed previously by Priebe and colleagues [11] used to sample the geographical regions across England from where the supported accommodation services were recruited for the national survey conducted during the QuEST study [3]. It provides a spread of scores on local authority areas on factors that influence mental health supported accommodation provision (mental health morbidity, social deprivation, degree of urbanisation, provision of community mental health care, provision of supported accommodation, mental health care spend per capita and housing demand); (2) service size (total number of service user beds/places per service); (3) staffing intensity (total full-time-equivalent (FTE) staff divided by total number of service user beds/places); (4) usual expected length of stay; (5) service user sex ratio (total number of males divided by total number of occupied beds/places) and (6) service user ability (number of current service users ‘generally able to do very little without assistance’ divided by the number of service users ‘generally able to do some things without assistance’ plus the number of service users ‘generally able to most things without assistance’).

Staffing intensity withstanding, these characteristics were selected as they have previously been shown to be associated with quality of care in inpatient mental health rehabilitation services [10, 12]. We included staffing intensity as we were aware this varies considerably between different types of supported accommodation services. We used the local authority area rank index [13] score rather than the previously used urban/rural dichotomous variable [12] as it provided a more comprehensive composite score of factors relating to location, including urban/rural setting. The other five variables are descriptive items collected during the completion of the QuIRC-SA (they do not contribute to any of the domain scores). These six variables were tested for multicollinearity and found not to be highly correlated.

All seven QuIRC-SA domain scores were normally distributed and were separately analysed as dependent variables using multiple linear regression models (thus creating seven models). Parameter estimates of the linear regression models were computed using robust clustered standard errors, with service type as the cluster variable (residential care, supported housing, floating outreach). Changes in domain scores per one unit increase for service variables with continuous data and their 95% confidence intervals (estimated using bootstrapping) are presented (local authority area index score, total beds/places and expected usual length of stay). For service variables that are ratios (staffing intensity, service user sex and service user ability), we present change in the domain score for one standard deviation (SD) increase in service variable.

## Results

### Missing data

Having an expected usual length of stay was missing for 13 of the 150 completed QuIRC-SAs (12 residential care and 1 supported housing services). It was assumed that this was most likely to be due to the service not having an expectation of service users moving on and thus no usual expected length of stay. Therefore, these missing values were replaced with the maximum value for this variable (20 years) prior to any analysis.

### Service characteristics

The total number of beds/places per service ranged from 3 to 80 with floating outreach tending to have larger services (mean 23 places) and supported housing having the fewest places per service (mean 11). Residential care services had the highest staff to client ratio (0.72), and floating outreach the lowest (0.17). The mean length of stay was longest in residential care services (mean 12.32 years) and lowest in floating outreach (2.83 years). The percentage of beds/places occupied by male service users was similar for residential care (70%) and supported housing (71%), and slightly lower for floating outreach (59%). Seven (24%) residential care and 19 (22%) supported housing services only accepted male service users. As expected, residential care services had a much higher proportion of residents able to do very little without assistance (28%), compared to supported housing (11%) and floating outreach (12%) services. Table 3 shows the service characteristics.

**Table 3:** Descriptive statistics of service variables, by type of service

Service variable	Residential care			Supported housing			Floating outreach			All types		
	N	Range	Mean (SD)	N	Range	Mean (SD)	N	Range	Mean (SD)	N	Range	Mean (SD)
Local authority area index score	28	−0.54, 1.78	0.91 (0.83)	87	−0.76, 1.96	0.90 (0.99)	35	−0.76, 1.78	0.62 (0.92)	150	−0.76, 1.96	0.84 (0.95)
Total beds/places	28	7.00, 40.00	19.46 (7.59)	87	3.00, 28.00	11.20 (5.20)	35	5.00, 80.00	29.97 (22.90)	150	3.00, 80.00	17.12 (14.35)
Staffing intensity (total staff FTE divided by total beds/places)	28	0.34, 2.25	0.72 (0.40)	87	0.10, 1.61	0.45 (0.27)	35	0.03, 0.97	0.17 (0.17	150	0.03, 2.25	0.43 (0.33)
Expected usual length of stay (years)	28	2.00, 20.00	12.32 (7.82)	87	1.00, 20.00	3.38 (3.43)	35	1.00, 9.00	2.83 (2.16)	150	1.00, 20.00	4.92 (5.63)
Service user sex ratio (percentage of male service users)	28	0.30, 1.00	0.70 (0.22)	87	0.00, 1.00	0.71 (0.25)	35	0.00, 0.90	0.59 (0.20)	150	0.00, 1.00	0.68 (0.24)
Service user ability (percentage ‘able to do very little’)	28	0.00, 1.00	0.28 (0.30)	87	0.00, 0.71	0.11 (0.18)	35	0.00, 0.50	0.12 (0.16)	150	0.00, 1.00	0.15 (0.21)

### Service quality (QuIRC-SA domain scores)

Supported housing scored higher than residential care on all seven of the QuIRC-SA domains, and higher than floating outreach on six of the domains. Floating outreach scored 88% on the Social Interface domain, the highest domain score by service type. On average (mean), the Social Interface domain was also the highest scoring out of all the domains across the service types (81%), and Human Rights the lowest (52%). Table 4 shows the mean and standard deviation of each QuIRC-SA domain score by service type and across services.

**Table 4:** QuIRC-SA domain scores, by service type

QuIRC-SA domain	Residential care (*n* = 28)		Supported housing (*n* = 87)		Floating outreach (*n* = 35)		All types (*n* = 150^a^)	
	Range	Mean (SD)	Range	Mean (SD)	Range	Mean (SD)	Range	Mean (SD)
Living Environment %	54, 96	77 (10)	62, 96	78 (8)	–	–	54, 96	78 (8)
Therapeutic Environment %	38, 72	57 (7)	41, 77	61 (7)	45, 71	59 (5)	38, 77	60 (7)
Treatments and Interventions %	32, 80	63 (11)	54, 84	69 (7)	58, 75	66 (4)	32, 84	67 (7)
Self Management and Autonomy %	30, 67	51 (9)	28, 85	55 (12)	38, 77	53 (9)	28, 85	54 (11)
Social Interface %	55, 96	76 (10)	58, 92	80 (8)	77, 97	88 (5)	55, 97	81 (9)
Human Rights %	39, 69	53 (7)	36, 78	53 (8)	35, 62	48 (6)	35, 78	52 (8)
Recovery Based Practice %	20, 86	61 (14)	47, 87	69 (10)	50, 77	66 (7)	20, 87	67 (10)

^a^Floating outreach services do not score for Living Environment, therefore 35 QuIRC-SAs are removed from the total sample of 150 for this domain

### Associations between service quality (QuIRC-SA domain scores) and service characteristics

Table 5 shows the estimated change in QuIRC-SA domain score per one unit increase in the service variable. Where the service variable is a ratio (staffing intensity, service user sex ratio and service user ability), the change in domain score per one SD increase in the service variable is also presented. Associations between service variables and domain scores with *p* values less than 0.05 are described below.

**Table 5:** Change in domain score per one unit/SD^a^ increase in service variable (95% confidence intervals) and p values, for each linear regresion model (domain)

Service variable	SD for ratio service variables^a^	Living Environment (*n* = 115)	Therapeutic Environment (*n* = 150)	Treatments and Interventions (*n* = 150)	Self-Management and Auton. (*n* = 150)	Social Interface (*n* = 150)	Human Rights (*n* = 150)	Recovery Based Practice (*n* = 150)
Local authority area index score	–	−2.3 (− 2.6, − 2.0), < 0.001	−1.8 (− 3.2, − 0.5), 0.007	− 1.4 (− 3.3, 0.5), 0.140	− 0.4 (− 1.5, 0.7), 0.512	−2.9 (−6.6, 0.7), 0.114	−2.2 (− 3.8, − 0.6), 0.006	−2.0 (− 2.9, − 1.2), < 0.001
Total beds/places	–	− 0.0 (− 0.4, 0.4), 0.908	−0.1 (− 0.1, 0.0), 0.001	−0.1 (− 0.2, − 0.1), 0.001	−0.1 (− 0.2, 0.0), 0.072	−0.1 (− 0.3, 0.0), 0.052	0.0 (− 0.3, 0.3), 0.996	−0.1 (− 0.2, 0.0), 0.043
Staffing intensity (total staff FTE divided by total beds/places)^a^	–	− 0.4 (− 1.5, 0.7), 0.474	1.5 (0.8, 2.2), < 0.001	6.0 (3.9, 8.1), < 0.001	1.4 (− 10.6, 7.8), 0.061	− 1.4 (− 10.6, 7.8), 0.767	− 6.1 (− 10.4, − 1.8), 0.006	1.3 (− 3.4, 6.0), 0.598
	0.3	−0.1 (− 0.5, 0.2), 0.474	0.5 (0.3, 0.7), < 0.001	2.0 (1.3, 2.7), < 0.001	0.5 (0.0, 0.9), 0.061	− 0.5 (− 3.5, 2.6), 0.767	− 2.0 (− 3.5, − 0.6), 0.006	0.4 (−1.1, 2.0), 0.598
Expected usual length of stay (years)	–	− 0.2 (− 0.4, 0.0), 0.017	−0.4 (− 0.5, − 0.3), < 0.001	−0.1 (− 0.2, 0.0), 0.019	−0.4 (− 0.8, − 0.1), 0.019	−0.3 (− 0.4, − 0.2), < 0.001	−0.3 (− 0.6, 0.1), 0.151	−0.7 (− 0.8, − 0.6), < 0.001
Service user sex ratio (percentage of male service users)^a^	–	− 1.5 (− 10.7, 7.8), 0.753	3.2 (− 1.3, 7.7), 0.166	5.9 (1.0, 10.8), 0.018	3.9 (−0.7, 8.5), 0.098	5.0 (−0.2, 10.3), 0.061	−2.5 (− 5.0, 0.0), 0.046	4.6 (−4.3, 13.5), 0.308
	0.2	−0.4 (− 2.5, 1.8), 0.753	0.8 (− 0.3, 1.8), 0.166	1.4 (0.2, 2.6), 0.018	0.9 (− 0.2, 2.0), 0.098	1.2 (− 0.1, 2.4), 0.061	−0.6 (− 1.2, 0.0), 0.046	1.1 (− 1.0, 3.2), 0.308
Service user ability (percentage ‘able to do very little’)^a^	–	− 0.1 (− 2.0, 1.9), 0.942	−1.5 (− 7.0, 3.9), 0.583	1.0 (− 8.3, 10.3), 0.83	− 3.7 (− 14.2, 6.7), 0.485	5.1 (− 5.8, 16.1), 0.357	−1.4 (− 8.2, 5.5), 0.691	−5.5 (− 13.1, 2.2), 0.160
	0.2	0.0 (−0.4, 0.4), 0.942	− 0.3 (− 1.5, 0.8), 0.583	0.2 (− 1.8, 2.2), 0.833	−0.8 (− 3.0, 1.4), 0.485	1.1 (−1.2, 3.4), 0.357	−0.3 (− 1.8, 1.2), 0.691	−1.2 (− 2.8, 0.5), 0.160

^a^For service variables that are ratios (staffing intensity, service user sex ratio and service user ability), the change in domain score per one SD increase in the service variable has also been included

### Living Environment

For this domain there were no ratings available for floating outreach services and therefore analysis was based on 115 rather than 150 services. The mean Living Environment domain score across all services was 78% (residential care 77%, supported housing 78%). Each one point increment in the local authority area index score was associated with a reduction in the Living Environment score of 2.3 percentage points. With each additional year of usual expected length of stay, the Living Environment domain score decreased by 0.2 percentage points (95% CI: -0.4 to − 0.0).

### Therapeutic Environment

The mean Therapeutic Environment domain score across all services was 60% (residential care 57%, supported housing 61%, floating outreach 59%). Each one point increment in the local authority area index score was associated with a reduction in the Therapeutic Environment score of 1.8 percentage points. Each additional bed/place was associated with a decrease in the Therapeutic Environment domain score of 0.1 percentage points (95% CI: -0.1 to 0.0). An increase in the staff to service user ratio of 0.3 (one SD) was associated with an increase of 0.5 percentage points in the Therapeutic Environment domain score (95% CI: 0.3 to 0.7). With each additional year of usual expected length of stay, was associated with a decrease in the Therapeutic Environment domain score of 0.4 percentage points (95% CI: -0.5 to − 0.3).

### Treatments and Interventions

The mean Treatments and Interventions domain score across all services was 67% (residential care 63%, supported housing 69%, floating outreach 66%). Each additional bed or place per service was associated with a reduction in this domain score of 0.1 percentage points (95% CI: -0.2 to − 0.1). Each increase in the staff to service user ratio of 0.3 (one standard deviation) was associated with an increase in the Treatments and Interventions domain score of 2.0 percentage points (95% CI: 3.9 to 8.1). With each additional year of usual expected length of stay was associated with a decrease in the Treatments and Interventions domain score of 0.1 percentage points (95% CI: -0.2 to 0.0). An increase of 0.2 (one SD) in the ratio of male service users to places was associated with an increase in the Treatments and Interventions domain score of 1.4 percentage points (95% CI: 0.2 to 2.6).

### Self-Management and Autonomy

The mean Self-Management and Autonomy domain score across all services was 54% (residential care 51%, supported housing 55%, floating outreach 53%). Each additional year of usual expected length of stay was associated with a decrease in this domain score of 0.4 percentage points (95% CI -0.8 to − 0.1).

### Social Interface

The mean Social Interface domain score across all services was 81% (residential care 76%, supported housing 80%, floating outreach 88%). Each additional year of usual expected length of stay was associated with a decrease in this domain score of 0.3 percentage points (95% CI: -0.4 to − 0.2).

### Human Rights

The mean Human Rights domain score across all services was 52% (residential care 53%, supported housing 53%, floating outreach 48%). Each one point increment in the local authority area index score was associated with a reduction in the Human Rights domain score of 2.2 percentage points. An increase in the staff to service user ratio of 0.3 (one SD) was associated with a decrease in this domain score of 2.0 percentage points (95% CI: -3.5 to − 0.6). An increase in the ratio of male service users to places of 0.2 (one SD) was associated with a reduction in the Human Rights domain score of 0.6 percentage points (95% CI: -1.2 to 0.0).

### Recovery Based Practice

The mean Recovery Based Practice domain score across all services was 67% (residential care 61%, supported housing 69%, floating outreach 66%). Each one point increment in the local authority area index score was associated with a reduction in this domain score of 2.0 percentage points. Each additional bed/place per service was associated with a reduction in this domain score of 0.1 percentage points (95% CI: -0.2 to 0.0) and each additional year of usual expected length of stay was associated with a reduction of 0.7 percentage points (95% CI: -0.8 to − 0.6).

Resident ability was not associated with any of the QuIRC-SA domains.

## Discussion

Supported accommodation is a key component of community mental health care for service users with more complex needs. Identification of service characteristics that are associated with better quality care is of obvious importance. The QuIRC-SA is a standardised quality assessment measure with good inter-rater reliability across a range of different service types.

Six of the seven service characteristics we investigated were associated with one or more of the QuIRC-SA domain scores; local authority area index score, service size (number of beds/places), proportion of male service users, staffing intensity, and the expected usual length of stay. The latter variable was negatively associated with six of the seven QuIRC-SA domains. The Local Authority index had the most influence on domain scores, with a one point increment being associated with a reduction of up to 2.3% in four of the QuIRC-SA domains. This multi-dimensional index includes markers of demand (urbanicity, psychiatric morbidity and housing) and investment (spend on mental health and supply of community based services). Salisbury and colleagues recently established the association between the amount spent on mental health in a geographic area and the quality of longer term care [14]. Our results appear to corroborate this at the local level, suggesting that local investment needs to respond to local demands to ensure adequate quality of care is provided to people with longer term and more severe mental health problems living in supported accommodation.

Staffing intensity was positively associated with two domains (Therapeutic Environment, Treatments and Interventions), and negatively associated with one of the domains (Human Rights). This is consistent with findings by Sandhu and colleagues [15], where adequate staffing was considered by staff and service users to be key to facilitating recovery. The negative association we found between this variable and Human Rights could perhaps reflect services with higher staffing having a more restrictive approach to supporting service users with more complex needs. In supported housing services in Canada, authoritarian staff management structures were found to have the least positive impact on services users where a “democratic, shared decision-making style” (p.1256) of staffing was preferred [16]. Additionally, Nelson and colleagues [17] report lower levels of staffing encouraged increased engagement with service users.

Increasing service size was negatively associated with Therapeutic Environment, Treatment and Interventions and Recovery Based Practice domain scores. This finding concurs with previous research [6, 10], suggesting that larger services tend to be more institutional and less able to offer an individualised, rehabilitative approach.

Our results on the proportion of male service users per service differs somewhat from a previous study showing a negative association between the percentage of male service users in an inpatient rehabilitation unit and quality [12]. We found that having a higher proportion of male service users was negatively associated with the Human Rights domain score but positively associated with the Treatments and Interventions domain score. This could be an artefact in that many male only supported accommodation services cater for service users who have a forensic history, offering specialist treatments (such as substance misuse interventions) in an environment which is necessarily more rule bound than other services since service users are often subject to legal restrictions and conditions associated with being permitted to live in the community.

We found no association between service user ability and quality of care. This concurs with findings from a national survey of inpatient mental health rehabilitation services [12]. This is important as service user ability can sometimes be cited by staff as a reason for being unable to deliver a high quality service.

### Strengths and limitations

The data analysed in this study were collected using a specialist, standardised service quality assessment tool for mental health supported accommodation services that has been shown to have good psychometric properties [9]. We used multilevel modelling for our data analysis to take account of clustering at the service type level. We agreed the variables that we would investigate for their association with quality of care prior to carrying out our analyses, choosing these on the basis of previous research. In addition, our sample size was adequate for our analyses. The sample included more supported housing services than the other two service types, in keeping with national provision [3]. However, our analyses used data collected for other purposes and not all the services were randomly selected (87 were randomly selected for the QuEST national survey). Furthermore, we only included services based in England and therefore the findings cannot be generalised to supported accommodation services in other countries.

### Implications

Whilst under resourcing of supported accommodation services can only be addressed at a political level, we have identified other factors that are associated with better service quality that could be incorporated into service planning. Having a shorter expected length of stay was associated with better quality services, presumably because it facilitates a more focused approach to individual goal setting with service users that can assist their recovery and help them gain the necessary skills to move on successfully to more independent accommodation (reflected in the Self-Management and Autonomy QuIRC-SA domain). This creates a positive and hopeful culture reflected in the Therapeutic Environment and Recovery Based Practice domains, and is not limited by general service user ability. However, adequate staffing is clearly essential to achieve this, a factor related to the size of the service. Larger service size was negatively associated with three of the seven QuIRC-SA domains. A balance therefore has to be struck between providing adequate staffing and a service size that is economically viable. Finally, services with higher male service user ratios fared better on quality, but single sex services will continue to be needed due to the challenges posed by individuals with certain types of risk.

## Conclusions

This study has helped to identify general service structures and characteristics that can drive up quality of care in supported accommodation services. Services should adopt an expected usual length of stay and be of a moderate size with adequate staffing to support service users to gain and regain skills for more independent living. However, the feasibility of such changes are likely to be constrained by resources and the nature of services; an expected usual length of stay and move-on to more independent settings might be less realistic for services to implement that provide high levels of support.

## Abbreviations

FTE: Full-time-equivalent
NIHR: National Institute for Health Research
QuEST: Quality and Effectiveness of Supported Tenancies for people with mental health problems
QuIRC: Quality Indicator for Rehabilitative Care
QuIRC-SA: Quality Indicator for Rehabilitative Care-Supported Accommodation
SD: Standard deviation
